# ‘Capacity for what? Capacity for whom?’ A decolonial deconstruction of research capacity development practices in the Global South and a proposal for a value-centred approach

**DOI:** 10.12688/wellcomeopenres.16850.1

**Published:** 2021-05-26

**Authors:** Maru Mormina, Romina Istratii

**Affiliations:** 1 Ethox Centre and Wellcome Centre for Ethics and Humanities, Big Data Institute, University of Oxford, Li Ka Shing Centre for Health Information and Discovery, Oxford, OX3 7LF, UK; 2 Department of Religions and Philosophies, School of History, Religions and Philosophies, School of Oriental and African Studies (SOAS), University of London, Thornhaugh Street, Russell Square, London, WC1H 0XG, UK

**Keywords:** research capacity development, low- and middle-income countries, science technology and innovation, knowledge production, cognitive justice, localisation, decolonial reflexivity.

## Abstract

Whilst North to South knowledge transfer patterns have been extensively problematised by Southern and decolonial perspectives, there is very little reflection on the practice of research capacity development (RCD), still strongly focused on technoscientific solutionism, yet largely uncritical of its underlying normative directions and power asymmetries. Without making transparent these normative and epistemological dimensions, RCD practices will continue to perpetuate approaches that are likely to be narrow, technocratic and unreflexive of colonial legacies, thus failing to achieve the aims of RCD, namely, the equitable and development-oriented production of knowledge in low- and middle-income societies. Informed by the authors’ direct experience of RCD approaches and combining insights from decolonial works and other perspectives from the margins with Science and Technology Studies, the paper undertakes a normative and epistemological deconstruction of RCD mainstream practice. Highlighting asymmetries of power and material resources in knowledge production, the paper’s decolonial lens seeks to aid the planning, implementation and evaluation of RCD interventions. Principles of cognitive justice and epistemic pluralism, accessibility enabled by systems thinking and sustainability grounded on localisation are suggested as the building blocks for more reflexive and equitable policies that promote research capacity *for *the purpose of creating social value and not solely for the sake of perpetuating technoscience.

## Introduction

Research capacity development (RCD), often also called *research capacity building* or *research capacity strengthening*, is defined as ‘ *an ongoing process of empowering individuals, institutions, organizations and nations to: define and prioritize problems systematically; develop and scientifically evaluate appropriate solutions; and share and apply the knowledge generated’* ( Lansang & Dennis, 2004: 764–5). In the context of international development, RCD refers to interventions, typically delivered by high-income countries (HICs), that seek to enhance the ability of individuals and institutions in low- and middle-income countries (LMICs) to produce and use scientific knowledge to solve social problems. Interventions range from doctoral and postdoctoral training programmes and grants for collaborative research to institutional twining arrangements for the sharing of resources and best practice. The practice attracts substantial investment from donor agencies, while in the context of the UK, it comprises a key component of research funding schemes, with the most prominent being until recently the Global Challenges Research Fund (GCRF).

RCD is predicated on a belief about the centrality of scientific knowledge in the development process. Since US President Truman’s famous speech in 1949 pledging to make ‘ *the benefits of our scientific advances and industrial progress available for the improvement and growth of underdeveloped areas*’, Science, Technology and Innovation (STI) has been at the heart of the development discourse. Cummings *et al.* (2018) call this centrality the technoscientific-economic paradigm (henceforth, the technoscientific paradigm). Its roots are in the Enlightenment ideal of modernity which considers development as a linear transition from the ‘backward’ subsistence economies encountered in LMICs in the so-called the Global South, to the ‘technologically advanced’ knowledge economies of HICs in the Global North (see for example Escobar, 1995; Esteva, 1992; Kothari *et al.*, 2019) ^1^. This view of development has shaped the relationship of western societies to their former colonies in the post-independence era. If STI is pivotal to economic prosperity, western societies have a duty to use their STI capabilities to solve the problem of underdevelopment in LMICs. This mindset gave rise to top-down, expert-driven models of technical assistance that were popular in the 1950s and 1960s but proved largely unsuccessful due to failing to involve substantively local stakeholders. Technical assistance was *imposed* upon local stakeholders, as opposed to being *owned* by them. By the 1990s, a new capacity development paradigm sought to redress these failures by replacing top-down, supply-driven approaches with demand-driven processes that emphasised local ownership and partnership and were more sensitive to different contexts and realities. This was a paradigm shift, arguably more in theory than in practice ( Venner, 2015), that challenged *how* development was done, yet not its premises. Indeed, the transfer of knowledge and technologies, whether through technical assistance or capacity development, continues to be at the heart of the western-inspired development project.

This paper views RCD as a continuation of colonial processes of homogenisation and cultural assimilation which, like the technoscientific model of development that inspires it, is presented as a global universal design that simultaneously downgrades other forms of knowing ( Dunford, 2017). This epistemic hegemony intersects with other hegemonies - economic, political, racial, gendered, cultural - to form a *matrix of power*, resulting in the appropriation of knowledge, extraction of natural resources, and destruction of non-western epistemologies and ways of being ( Hall & Tandon, 2017; Harding, 2003; Smith, 1999). In challenging allegedly universal, yet narrowly defined, western ideas of development as technology acquisition as the premise of RCD, it is possible to promote epistemic diversity as called for historically by decolonisation movements and critiques. Embracing pluralistic epistemologies, which we understand as ways of knowing and producing knowledge ( Istratii, 2017; Ladson-Billings, 2000), is necessary in order to move away from the current paradigm whereby the West continues to define standards for the rest of the world.

Decolonial theory ( Mignolo & Walsh, 2018; Quijano & Ennis, 2000) offers a useful lens to critically examine current approaches to RCD and reveal how these remain rooted in a technoscientific narrative of development that, while presented in apolitical, value-neutral terms, in fact obscures the normativity of colonial legacies and ongoing dynamics. Using a decolonial lens, we argue that, while predicated on the need for robust local research systems, RCD perpetuates prevailing western worldviews and homogenising approaches to knowledge production through the export of western-inspired notions of *good* or *desirable* knowledge. Ultimately, RCD shapes what knowledge is produced (and which is left unspoken), how it is distributed, to what social groups and what conceptions of development it promotes.

The reflections that follow are informed by our combined experiences in researching the politics and ethics of development-oriented research (MM), supporting international partnerships funded by GCRF (MM, RI) and community-based work to promote locally grounded ways of understanding and addressing development-oriented issues in Southern contexts (RI). We are also influenced by our experiences of working at two different UK institutions with salient colonial histories. These combined positionalities allow us to bring together decolonial critiques and insights from Science and Technology Studies to contribute an analysis of RCD that deconstructs its premises and reveals latent tensions between attempts to shift power and achieve greater localisation of knowledge production processes on one hand, and enduring Eurocentric definitions of research capacity that perpetuate the hegemony of and therefore dependency on Northern expertise, on the other.

A critical revision of RCD praxis can deepen our appreciation of this inherent contradiction and help foster alternative approaches grounded in a decolonial ethos. One such alternative approach is proposed here. It has *cognitive justice*, *systems thinking* and *localization* as its building blocks and *social value* as its explicit aim. *Cognitive justice* is an ethical principle that asserts the diversity of knowledges and equality of all knowers ( Visvanathan, 1997), and can help ground more nuanced measures of capacity that counter Northern-centric, positivistic and homogenising understandings of knowledge production. *Systems thinking* underpins various frameworks that attempt to explain processes of knowledge creation and mobilisation such as the *National Innovation System* ( Lundvall, 1992) and the Sabato Triangle ( Sabato & Botana, 1968). These frameworks are primarily concerned with the *whole* and its relationship to its context. They conceptualise knowledge systems as webs of interrelations and patterns of interactions that take place within specific socio-political, economic and cultural contexts and shape how knowledge is created and translated into social value (products, processes or services). Applying systems thinking to RCD allows overcoming capacity analyses that remain a-historical, apolitical and a-cultural, and focused on bringing top-down and western-centric solutions without attention to political and socio-economic contexts or to how networks of social relationships can unleash or impair capacity. *Localisation* in the context of RCD refers to the process of recognising, respecting and strengthening the leadership by local actors and their capacity to determine their own knowledge needs in order to better address their self-defined development priorities. An approach based on these three pillars ultimately aims to unsettle unquestioned assumptions and shift RCD praxis towards supporting more equitable, holistic and grounded knowledge production processes that do not reproduce conditions of injustice but advance social value. *Social value* here is understood as a positive impact to the equitable long-term wellbeing and resilience of individuals, communities and wider society, where positive impact is determined by context-specific conditions and dynamics, institutions, traditions and worldviews.

The paper argues that research capacity exists in the Global South but in order to *see* it and adequately support it, donors and research partners must be able to embrace epistemic diversity. This would shift the linear, technocratic, metric-based and context-insensitive nature of interventions to messier but more context-specific, holistic (i.e. systemic) approaches to RCD that support truly localised processes of knowledge production that create social value. An approach to RCD built on cognitive justice, systems thinking and localisation, with social value at its heart should be concerned with:

a) promoting processes of knowledge production that can foster *epistemic pluralism* and diversity of experiences and priorities;

b) making the benefits of knowledge more *accessible* in view of social inequalities by considering the system of knowledge production and utilisation as a whole; and

c) fostering knowledge production processes that can be *sustained* independently over time because they are locally grounded.

Supporting research capacity for social value requires constant and reflexive engagement with questions, such as: ‘capacity for what and for whom?’ during the design, implementation and evaluation of all RCD efforts. Reflexivity is needed to pursue effective approaches to knowledge production that are holistic and adaptive, do not ignore power dynamics but give attention to the political root causes of capacity gaps, and engage those it seeks to benefit in setting priorities to avoid and reverse current trends of dictating outcomes.

## The technoscientific paradigm of development and its relation to RCD

Since the 18 ^th^ century, the idea of science as a driver of social change has underpinned national development efforts, as Western Europe’s industrialisation experience demonstrates. The notion of *knowledge society* or *knowledge economy*, popularised in the 1960s and 1970s ( Drucker, 1968; Machlup, 1962), linked STI to economic development, especially during the post-war years, in both the domestic and foreign policies of most industrialised nations ( Hornidge, 2011). This is a form of technological determinism (the technoscientific paradigm) by which development follows a linear progression from technology adoption to economic growth ( Cherlet, 2014; Cummings *et al.*, 2018). It has been championed by development agencies, particularly the World Bank ( Broad, 2007; Enns, 2015) as well as within the context of the Sustainable Development Goals (SDG) ( Cummings *et al.*, 2018).

STI undoubtedly contributes to development. Advances in medical and other technologies have enabled many of the inroads made in the last century (such as reduction in maternal and infant mortality and antiretrovirals in the fight against malaria and HIV). However, the promotion of the technoscientific approach is not a value-neutral process. Knowledge production is inseparable from positionality and geography and, therefore, the dominance of the technoscientific paradigm implies the dominance of the worldview that has generated it. Appraised from this perspective, the technoscientific paradigm serves a universalising, deterministic, teleological, western, and inevitably westernising, narrative of development that downgrades other forms of knowledge (see for example Escobar, 1995 for a fuller critique of this model of development; on the universalising and teleological tendencies of development see the more recent critique advanced in Istratii, 2020).

The technoscientific paradigm, favoured by 20 ^th^ century globalisation, has become so dominant that it is impossible to think of development outside of its relation to STI. According to this paradigm, to participate in the global marketplace, to lift citizens out of poverty LMICs must *catch up* with industrialised nations by becoming knowledge economies. This then creates the need for RCD, attested in the large body of literature devoted to it. Most of this literature is found within the health sciences, but also within the fields of climate/environment, agriculture, and information, communication and technology (ICT). A literature search on Google Scholar using the terms *research capacity* AND *building* OR *development* OR *strengthening* returned 49,200 articles published since 2000; when the same search was conducted, excluding the words *health*, 16,900 articles were returned; lastly, when *technology*, *agriculture*, *agricultural*, *climate*, *environmental* and *ICT* were also excluded, the number of articles returned dropped to 3,320. Whilst this is only a crude scientometric analysis, it sufficiently evidences that RCD has been predominantly directed towards strengthening the production of knowledge relevant to the knowledge economy.

A review of the burgeoning RCD literature in these fields suggests an acceptance of the technoscientific paradigm’s premises that is unquestioning and uncritical. RCD is generally discussed in evaluative terms to draw best practice. If challenges are identified, they mostly relate to implementation issues, for example, language barriers, authorship arrangements, differing expectations, expertise, interest and agendas, limited institutional and political commitment to research, political or economic instability, or other (see Vasquez *et al.*, 2013 for a review of the literature on RCD). What is not immediately evident in these studies is reflexivity about the universalising premises of the technoscientific paradigm that are replicated in RCD interventions and which preclude consideration of the wider context and epistemological frameworks underpinning scientific knowledge production. For example, an intervention may seek to improve the skills of LMICs PhD supervisors, but it may unreflexively model the intervention on the (assumedly universal) expectations and roles of PhD supervisors in western institutions, failing to see how context-specific, cultural or educational factors affect student-supervisor dynamics ( Madsen & Adriansen, 2019). The strong focus on the transfer of technical knowledge and skills obscures the fact that RCD is, after all, a social intervention that affects the lives of real people. Reflecting on the unexamined assumptions upon which interventions are built is urgently needed to minimise some of the more negative unintended consequences.

## A decolonial critique

The aforementioned approach to RCD, rooted in a technoscientific model of development assumed as normative and universal, and lacking sufficient attention to wider social contexts, follows the seemingly apolitical technocracy of current development discourses ( Vessuri & Cancino, 2019). These technocratic framings and approaches to RCD emphasise transfers of knowledge, methods, technologies or research processes, presented as politically and value-neutral. This, however, overlooks the underlying and enduring *colonial matrix of power* ( Quijano & Ennis, 2000), constituted of intersecting hierarchies -political, economic, cultural, racial, gender-related and epistemic- and which is reinforced through RCD interventions. This is because the transfer model that underpins these interventions presupposes a *deficit*, thus replicating colonial power hierarchies that portray the South as the location of problems and backwardness and the North as the location of solutions and progress. The coloniality that imbues RCD is, sadly, still poorly acknowledged in mainstream practice. RCD’s faith in the power of STI to lift the yoke of underdevelopment without due attention to the cultural context or socioeconomic realities in which knowledge production takes place parallels the colonist’s faith in the superiority of western science and implies the devaluation and silencing of other knowledges and experiences.

Science, in most non-western contexts, is ‘ *inextricably linked with European colonialism*’ ( Smith, 2004: 1); it helped justify the *civilizing mission* and continues to reinforce the epistemic boundaries between a developed centre and the developing peripheries. Central to the colonial project was the erosion of indigenous knowledge systems and the imposition of a superior epistemology as a means of social control and exploitation of human and natural resources ( Fanon, 2001 [1961]; Istratii & Hirmer, 2020; Smith, 1999). This was a form of epistemicide or killing of knowledge systems ( Grosfoguel, 2013), a cognitive injustice ( de Sousa Santos, 2015) whose legacy is the South’s lack of intellectual self-confidence ( Neylon, 2019) and its dependency on the knowledge of the Global North for the solution of problems. It is no coincidence that much RCD is driven by *experts* from HICs. Western knowledge may no longer be overtly framed as superior, yet RCD still promotes a development imaginary that has western science as its key referent.

It may be argued that RCD seeks localisation by empowering researchers from LMICs to lead in global knowledge production, and in this way destabilise some of the implied hierarchies. This is paradoxical, since empowering LMIC researchers and institutions to participate in the global market of ideas entails developing their capacity to conform to global (western) standards of research excellence that, ultimately, help maintain the hegemony of HICs as centres of knowledge production: publications in international high-impact journals, use of English as lingua franca, dictation of meaning of impact in research, etc. In other words, developing research capacity entails developing the ability to sustain scientific activity that adheres to particular standards of excellence, typically measured by quantitative proxy indicators of quality and impact. This notion of excellence underpinning RCD is hegemonic and homogenising because it inevitably configures and appraises research capacity in the Global South according to the metrics and standards of the Global North. Appraised in this way, knowledge production activities in LMICs are inevitably *insufficient* or *poor quality*, thus justifying the RCD industry. Framing capacity in terms of narrowly defined excellence leads to equally narrow approaches to RCD that value particular types of science, usually in support of some predefined development agenda, and thus continue western epistemic dominance.

Construed as a technocratic approach, RCD depoliticizes global epistemological inequalities by casting them as *technical capacity gaps*, thereby obscuring the more structural conditions that first created and maintain these gaps. Beneath the seemingly rigorous and politically-neutral language of *excellence* are normative assumptions about what constitutes good science, how it contributes to development, and who defines it ( Noxolo, 2017). These assumptions shape decisions about what research is funded and published, and consequently which disciplines, knowledges and capacities are promoted or marginalised, as we discuss in the example below. This has implications for how knowledge is used to define problems and shape policy, affecting a host of social, economic and environmental issues. In other words, assumptions about what constitutes good science shape the kinds of knowledge and capacities that are fostered and ultimately determine the trajectories of development.

Particularly relevant here is the role of donors, publishers, and academic institutions ( Demeter, 2020; Istratii & Lewis, 2019), which determine where and how research capacity is developed through the availability and priorities of funding, and preferences for certain topics, methodological approaches or disciplinary traditions. In promoting particular notions of excellence, these actors shape not only what science is produced and published, but also what capacities are needed to produce it.

An illustrative example can be drawn from Makerere University, the largest and oldest Ugandan university. The University, which received USD 214 million from foreign donors between 2000–2012, mainly for research, has a research portfolio heavily tilted towards the health sciences (particularly Immunology and Microbiology), as evidenced by the fact that more than 40% of its research outputs are in these areas ( Ssembatya, 2019). Its College of Health Science has the specific mandate to engage with research topics of interest to the international community. In contrast, research in Agriculture and Biological Sciences accounts for 12% of total outputs, despite the fact that agriculture accounts for 25% of Uganda’s GDP and employs 40% of the workforce ( *ibid*). The implication is that reliance on foreign funding and the need for visibility in international (HIC-based) publishing platforms pushes LMICs to skew their research portfolios and capacities towards foreign priorities, which may or may not have relevance locally.

We must acknowledge, however, that diffusing or challenging the hegemony of HICs, both in terms of definitions of quality and the epistemology in which research agendas are articulated, may not necessarily lead to approaches that promote desirable development in LMICs. While localisation (agency to define one's own capacity needs, research priorities and definitions of *valuable* knowledge) is necessary, is it sufficient to promote approaches to knowledge production that are pluralistic and create social value? The essentialisation of the *local*, often conceptualised in opposition to the *international* requires greater critical reflection to draw attention to who defines the local, who claims to represent it and how this may lead to the exclusion of certain voices and perspectives ( Roepstorff, 2020). Localisation of knowledge production should not be seen uncritically as a panacea but must engage seriously with questions of legitimacy and representation, often entwined with complex issues of class, privilege and various markers of group identity. Global asymmetries often replicate locally through exclusionary systems that disproportionately concentrate resources into ivory towers inhabited by homegrown elite classes, classes that have often benefitted from and assimilated the prevailing western narratives through RCD programmes.

The increased trend in recent decades towards creating *local* cadres of western educated researchers who have assimilated western ideas of progress raises the urgency for critical engagement with the premises of RCD, particularly in view of the impact that local adaptations of the technoscientific paradigm can have for societies. In decolonial discourse, RCD promises the localisation of knowledge production but without fully changing the *locus of enunciation* ( Mignolo, 1993), that is to say, the ideological and epistemological positions from where researchers construct knowledge. Simply changing the geographic locus of articulating research agendas but without unsettling the assumptions, definitions and power structures that condition how knowledge is created, shared and utilised is unlikely to offer a path towards equitable and sustainable research systems.

To be clear, we are not arguing for a rejection of modern western science or saying that indigenous knowledge should suffice to solve complex problems, especially when many such problems are shared cross-culturally (climate change, food security, pandemics, etc.). Addressing these problems effectively requires knowledge exchange and collaborative approaches. We are also aware that often oppressive practices can be framed in reference to indigenous traditions and normative systems, which should not be approached uncritically either. A more sensible approach would require an ethic of knowledge coexistence and complementarity that seeks to improve our understanding of the world by using ‘two eyes’ ( Bartlett *et al.*, 2012) but without denying that tensions and contradictions exist in some areas between indigenous and western epistemologies ( Broadhead & Howard, 2021). In those areas, a false presumption of equal validity can compromise genuine dialogue. This requires both moral judgement and evidence-based, people-centred research that understands lived realities and how they are affected by different epistemologies. Decolonising RCD does not mean a lesser focus on excellence/quality, either, but a contextualisation and diversification of knowledge production systems through cognitive justice and direct and substantive engagement with diverse actors in the communities of interest, who should not be limited to western-trained or elite-class researchers and development agents.

These observations point to the need for more nuanced discussions about RCD beyond dichotomous discourses: western vs indigenous or local vs international. If RCD is to support effective research systems that can practically improve people’s lives, it must upend its epistemological foundations rooted in a technoscientific solutionism that is exported as capacity development and judged according to western defined standards of quality. This will not be achieved by simply developing research systems and cadres of researchers that articulate locally western-inspired paradigms but by embracing a different, more situated conceptualisation of capacity that reflects on and challenges colonial legacies and begins with an articulation of knowledge production ‘ *oriented towards defending localised life-projects and life spaces’* ( Conway & Singh, 2009).

## Towards a new value-centred approach to RCD

The preceding sections outlined a critique of approaches to RCD based on the transfer of a technoscientific model of development that is reflective of post-colonial and neo-colonial divisions between knowledge production centres and peripheries (with the understanding that centres exist within peripheries and peripheries within centres). RCD reproduces many of these inequalities, despite the elusive rhetoric of partnerships, equity and ownership ( Beran *et al.*, 2017) imbued in its jargon. By presenting ‘underdevelopment’ in apolitical terms as a scientific and technological deficit, RCD obscures, in effect, its normative and political dimensions.

The assumption that the transfer of forms of knowledge accepted as standard in HICs propels economic development and social wellbeing internationally has been already exhaustively challenged. However, no systematic critique appears to have been produced in relation to RCD, specifically. Advocates of RCD in LMIC contexts often do so on the easily accepted assumption that scientific knowledge instrumentally contributes to overall economic growth and wellbeing ( Cozzens, 2007). However, the history of modernity, constructed largely on the basis of technological progress, shows that the development project (with STI at its heart) has, arguably, exacerbated inequalities between and within countries. The application of modern universal scientific solutions has simultaneously facilitated extractive, exploitative and destructive relationships with nature and with one another, and delegitimised indigenous knowledges that emphasise harmony with nature and the community ( Harding, 2003; Shiva, 1991). The answer to underdevelopment is not necessarily more technoscientific fixes but a broader view of science, one that provides multiple vantage points to understand the world and change it. This in turn requires a different approach to RCD. 

In what follows, we propose cognitive justice, system thinking and localisation as the pillars upon which new approaches to RCD may be envisaged. Such approaches entail a reflexive practice that engages more fully with the question of what purpose research capacity serves and who it benefits (‘capacity for what? capacity for whom?’). The starting point is an articulation of social goals and an explicit commitment to knowledge production that creates social value (understood as a positive impact to the equitable long-term wellbeing and resilience of individuals, communities and wider society). RCD interventions shaped by a reflexive commitment to justice and the creation of social value may better support processes of knowledge production that: promote *cognitive justice* and *epistemic pluralism* and does not uncritically prioritise the needs of certain groups (international, national or local); can make research capacity benefits more widely *accessible* through holistic, *system-wide strengthening*; and contribute to the development or strengthening of *localised* and therefore more *sustainable* knowledge systems that have the ability to function independently of external funding restricting their continuity and productivity over time.

### Moving towards cognitive justice and epistemological pluralism

RCD should be first and foremost a process that respects the agency and self-determination of diverse actors in society. Yet, enabling agency and ownership of the research process, whilst necessary, it is not sufficient to address the problems discussed since it may not lead to greater equity, especially if the epistemology in which this research capacity is premised remains embedded in western notions of valid and beneficial knowledge. An RCD model that is purposeful and socially valuable needs to engage with the idea of epistemological pluralism or *pluriversality* ( Kothari *et al.*, 2019), an active embrace of diverse knowledges and epistemologies.

Epistemological diversity requires understanding context-specific socioeconomic, political and cultural dynamics to ensure that the development of research capacity can proceed in locally-grounded ways. Structures, checks and balances and eventually mutual agreement and understanding need to be in place to avoid capacity-building favouring a limited elite class or skewing social benefits. A practice informed by pluriversality and cognitive justice should pay careful attention to issues of power and privilege in the production of knowledge and its application/translation for social benefit. This may mean, for example, widening access to research careers by offering opportunities to underrepresented groups. To achieve this, it may be necessary to reconfigure RCD programmes (for example, doctoral and postdoctoral fellowship schemes) to ensure that different types of talents are recognised and encouraged and not merely those that map onto western-conceived ideas of excellence, which can only be developed by already privileged groups fortunate to access international educational opportunities abroad.

Cognitive justice may also require counterbalancing the oft-seen concentration of research investments in a few *centres of excellence* -teams or institutions that concentrate expertise around particular topics- with more diversified investments ( Franzen *et al.*, 2017). Whilst the existence of centres of excellence arguably creates critical mass and confers national and international visibility in specific research areas ( Hellström, 2017), concentrating research capacity in a few high-performing institutions can restrict cognitive diversity and growth from below. It can also lead to short-termism and risk averse research, if institutions strategize their research activities motivated solely by the need to maintain their *excellence* status. All this can compromise social relevance and the ability to harness research with society-oriented outcomes. Pluriversality can suggest alternative investment strategies to achieve diversification (both geographic and epistemic) and greater diffusion of benefits in diverse societies.

A practice of RCD underpinned by a commitment to cognitive justice should also be concerned with strengthening the capacity of non-research actors to engage in public deliberation about the place of science in society and its role in development. In practice, this could mean extending the purview of RCD to communities and civil society organisations, policy makers, knowledge brokers, science communicators (publishers, journalists), and industry – in order to ensure:

a) Diverse definitions of research agendas and capacity development strategies that respond to the needs of all relevant stakeholders and avoid skewing research towards a narrow set of problems or priorities;
b) Inclusive and pluralistic engagements in the process of research and knowledge production so that research excellence achieves social relevance and considers socio-cultural norms and standards;
c) Inclusive and pluralistic engagements in the translation of research into practical applications, so that such applications can maximize wellbeing for those who are in direct need.

### Promoting equitable access to the benefits of research capacity through systems strengthening

RCD is predicated on its potential to reduce the knowledge gap *between* HICs and LMICs. However, the possibility that it might, in fact, widen inequality *within* LMIC societies through an uneven distribution of research benefits is rarely acknowledged. The mainstreamed technoscientific paradigm can reinforce unequal social relations, many of which already reflect colonial legacies, if its promises of economic growth and wellbeing are not accessible to segments of society that are most in need. Technoscientific advances often benefit certain groups more than others, usually those already favoured by the system ( Cozzens & Kaplinsky, 2009). For example, in the context of increased integration and globalisation facilitated by internet technologies, LMICs have benefitted from the outsourcing of labour from HICs. However, these opportunities have generally accrued in the middle and upper classes who, often due to their westernised education and exposure, can access and qualify for the job criteria defined by western standards. The complex relationship between technology and inequality suggests that developing research capacities in LMICs cannot be done without considering equity of access to the benefits of STI, both direct (products and services) and indirect (e.g. employment opportunities). Yet, equity of access must be understood in the context of historical patterns of advantage and disadvantage as specific to each society.

Strengthening research capacity is not a neutral process since through the innovations it facilitates, it creates winners and losers, as the lessons of the Green Revolution remind us ( Harwood, 2018; Patel, 2013; Shiva, 1991). Whilst its effects are still debated, the deployment of capital-intensive agriculture, particularly in India and Pakistan, to increase food grain production during the 1960s and 1970s had profound consequences upon vulnerable communities ( Patel, 2013). Mechanized farming, large-scale irrigation, chemical fertilizers, and high-yield seed varieties where not technologies designed with the poor in mind: they required access to capital and good quality land, which were only accessible to and controlled by wealthier farmers ( *ibid*). This resulted in differential adoption (at least initially) and negative externalities for poor rural communities, such as land displacement and increased social differentiation.

Similarly, several studies have suggested that health innovations may in some cases widen rather than reduce health disparities ( Chang & Lauderdale, 2009; Glied & Lleras-Muney, 2008; Korda *et al.*, 2011; Weiss *et al.*, 2018) as a function of both access and use ( Goldman & Lakdawalla, 2005). People with more social, financial or educational resources are more likely to access and harness medical advances ( Phelan & Link, 2005; Weiss & Eikemo, 2020). Costly new drugs or medical treatments create disparities of access for poorer individuals, regions or countries ( Iyengar *et al.*, 2016). For example, access to assisted reproductive technologies (ART) in LMICs remains limited to the more affluent sectors of society, with these technologies usually being offered in private clinics and at costs prohibitive to most people ( Chiware *et al.*, 2021). However, new technologies can also provide cost-effective health interventions. For example, increasing evidence shows that the use of mobile technologies for health (m-health) can improve access gaps in LMICs ( Beratarrechea *et al.*, 2014) ( Ngaruiya *et al.*, 2019).

Of course, the effects of the Green Revolution are contested ( Harwood, 2018; Patel, 2013), and the issue of unequal access to medicines and new technologies is complex, with significant variations across different socioeconomic and political LMIC contexts ( Weiss & Eikemo, 2020). Whilst generalisations are not warranted, these examples nonetheless help to draw attention to the important regulatory role of the state. For instance, in the case the Asian Green Revolution, state subsidies (for credit, fertilizer, and irrigation) and public investments infrastructure were crucial to facilitate adoption of new technologies by small farmers ( Djurfeldt *et al.*, 2005; Fan *et al.*, 2008) and to offset falling prices due to overproduction. In the case of health innovations, though the evidence is scant, the positive impact of m-health technologies documented in some studies may be attributed to them being embedded within public health programmes and, therefore, being subject to an inevitable degree of state intervention, as is the case when these technologies are delivered through public-private partnerships (for an example of such intervention see van der Merwe *et al.*, 2020). In contrast, ART are largely delivered within the private sector and are, therefore, more vulnerable to market forces of supply and demand. The degree of access (however defined) therefore correlates to the alignment or misalignment of technological innovations with public policy. A lesson that can be drawn from the above examples is that ensuring equitable access to the benefits of research cannot be done without adequate checks and balances. This calls for systemic approaches to RCD that strengthen not just the institutions of knowledge production (e.g. universities) and knowledge translation (e.g. industry) but also the governance structures to ensure that knowledge is not only produced but also applied and made accessible equitably.

The idea of systemic capacity harks back to the concept of *innovation systems* ( Freeman, 1989; Lundvall, 1992), and before that the theoretical model of Latin American theorists Jorge Sabato and Natalio Botana ( Sabato & Botana, 1968), known as the *Sabato triangle*. These models do not focus just on knowledge production but rather on innovation, namely the process of creation and application of knowledge to achieve a desired economic or social goal. Innovation is conceived in a systemic and relational way, as the product of dynamic interactions between organisations (universities, industry) and the institutions (norms, policies and practices) that govern the nature and extent of interactions and bring knowledge into use. Whilst a detailed discussion is beyond the aims of this article, central to these models is the idea that the relative strength of each part of the innovation system is less important than the connections among them. An approach to RCD underpinned by the technoscientific model of development that focuses exclusively on strengthening knowledge production but fails to strengthen also the institutions that facilitate the translation of research capacity into tangible benefits and their fair distribution in society, will most likely not serve the majority. A systems approach to RCD has been repeatedly called for on the grounds of effectiveness ( Franzen *et al.*, 2017; Khisa *et al.*, 2019), and we would argue also on the basis of equitable/diversified access. Such an approach should embrace a multidimensional conceptualisation of capacity as entailing capabilities and competencies that are both scientific and non-scientific. It should enhance linkages between the producers and the users of knowledge and the institutional frameworks that enable a fairer diffusion and distribution of such knowledge.

### Supporting localised and sustainable research systems

Roughly defined, sustainability is the ability to function over time and under all circumstances. In the natural world, sustainability refers to the natural equilibrium of ecosystems. In the social world, sustainability is the ability to meet the diverse needs of existing and future communities with efficiency and at an appropriate scale ( Daly, 1992). It is about ensuring personal and social wellbeing over time. In this sense, sustainability is, first and foremost, a matter of just distribution. Research capacity is sustainable when researchers and institutions can function independently over time to sustain scientific productivity and produce high-quality knowledge that contributes to long-term social and economic wellbeing ( Kahwa *et al.*, 2016). Sustainability, thus, relates to concepts of *continuity* and *productivity* and is integral to many of the commonly cited definitions of RCD ( Dean *et al.*, 2017).

Whilst RCD is predicated on and legitimized by a sustainability logic, funding availability and funders’ commitments do not reflect the rhetoric of continuity. RCD projects are subject to funding cycles and are consequently focused on short-term impact that can be achieved within the lifetime of a grant. This project-oriented approach fosters fragmentation, duplication and lack of coordination ( Nuyens, 2005) and has been repeatedly reported as inadequate ( Boyd *et al.*, 2013; Cole *et al.*, 2014; Franzen *et al.*, 2017; Neylon, 2019). This paper argues for an RCD process that is holistic, purposeful and guided by the objective to achieve social value for diverse actors and stakeholders. This cannot be achieved on the basis of a linear pre-defined theory or programme, but requires flexibility, adaptability and, most importantly, time. Such a realistic approach requires long-term availability of funding, resources and human capital to accompany and support this bumpy journey. More importantly, it requires re-strategizing how research funding is administered and allowed to flow from HICs, where most funders are based ( Istratii, 2020), so that decisions about knowledge production and utilisation are made locally and in alignment with countries’ own desired paths to development. This must go hand in hand with increased local financing, as we argue below.

With regards to productivity, it is important to revisit how the concept is defined and used. Scientific productivity is still appraised from a ‘deficit’ view of LMICs as places of scientific and technological disadvantage. This is because RCD’s focus on a narrow set of cognitive, rigidly specified and measurable skills renders invisible and devalues a rich array of skills, abilities and knowledge that abound in any context, including LMICs ( Wendland, 2016). The current quest for sustainability, understood as productivity, favours a scientific monoculture that commodifies knowledge and often fails to recognise that knowledge creation can take many forms. Knowledge creation does not follow a pre-specified blueprint but is an intrinsically valuable process of capability expansion that affords societies critical thinking to challenge the status quo and imagine their own path to long-term social and economic wellbeing ( Mormina, 2019). Sustainability, thus, should be about shifting power and epistemic agency to enable the pursuit of different development imaginaries.

Broader understandings of sustainability as power shifting would also discourage scientific productivity based on *isomorphic mimicry* ( Pritchett *et al.*, 2010), namely the adoption of western knowledge production systems perceived as efficient. Emulating systems of research governance and paradigms of excellence arguably successful in HICs may be inequitable in LMICs. For example, systems of cash incentives for researchers to publish in high quality journals have a long history in HICs ( Fulton & Trow, 1974) and lately have been also adopted by institutions in LMICs as a cost-effective mechanism to boost research productivity, despite suggestions that this encourages the perverse culture of *publish or perish* and associated dubious practices ( Kana, 2016). Consequently, a staple of RCD interventions is academic writing and publishing workshops that seek to maximize the opportunities of LMIC researchers to reach international audiences through publications in high-impact journals. Whilst such interventions may be motivated by a legitimate desire to redress the paucity of Southern researchers’ presence in global publishing fora ( Chan *et al.*, 2011) and to maximize opportunities for upward mobility within research professions, it legitimizes the mimicry of market-oriented processes of research governance that can be detrimental in contexts characterized by limited public financing. Without adequate local financing, a culture of publish or perish legitimized by Western-led RCD interventions can also push researchers to *follow the money*, as suggested in the previous example of Makerere University. The paradox of the current RCD practice is that by empowering LMIC researchers to participate in the global marketplace of science and ideas, it simultaneously subjects them to the pressures and incentives of an unequal global knowledge system dominated by western norms and standards.

An approach to RCD that discourages isomorphic mimicry would also move away from so-called indicators of excellence as sole measures of productivity and, instead, focus on social value as a measure of capacity. Fostering systems and processes required to produce locally-grounded and socially-valuable knowledge would disincentivise research on topics considered of global relevance and perceived to attract international funding and publishers’ attention. This could also gradually begin to redress the existing bias towards participation in international networks (whereby research priorities are determined in centres based typically in HICs and often with less than equitable terms of engagement), at the expense of national or regional research collaborations ( Bradley, 2016). Appraising research systems in terms of their ability to produce social value would also reduce pressures to publish in high-impact, paywalled and English-speaking international journals ( Demeter, 2020; Demeter & Istratii, 2020). Such pressures currently discourage local knowledge dissemination and uptake, reducing the potential of research to have meaningful social impact.

From this follows that sustainability goes hand in hand with localisation. This may necessitate decreasing LMICs’ reliance on international funding and enabling local agency through increased national spending in research and development (R&D). Such a proposal may be considered unrealistic given most countries’ competing and often pressing spending demands and strained public finances. Whilst space precludes thoroughly countering this argument, suffice to say that how much a country invests in R&D needs not always correlate with the size of its economy. One may compare Tunisia, a lower-middle income country, against Uruguay and Paraguay, two high- and upper middle- income countries, respectively, by World Bank classification. In 2016, Tunisia ( GDP per capita PPP $10,359) spent 0.6% of its GDP on R&D whilst Uruguay ( GDP per capita PPP $20,669) spent 0.4%. In the same year, Paraguay, ( GDP per capita PPP $12,029) spent 0.11% of its GDP on R&D, six times less than Tunisia. In contrast, both countries had similar levels of expenditure across other areas, such as health (6.99% of GDP in Tunisia vs 6.7% in Paraguay), whereas Uruguay spent considerably more on health (9.4% of GDP) than on R&D ^7^. The implication is that whether countries decide to invest in the long but expensive process of building national capacity to solve problems or continue relying on imported knowledge and consultancies is ultimately a political decision and not always an economic one. In many LMICs scientific research receives little political or social support ( Niosi, 2010; Sutz, 2003). Consequently, there is a weak evidence-based decision making culture ( Newman *et al.*, 2012; Stewart *et al.*, 2018) and little demand for knowledge, but fundamentally an absence of mechanisms for public deliberation about science’s place in society ( Dagnino, 2012). This brings us back to the importance of civic deliberation and the need for approaches to RCD, that because underpinned by a commitment to localisation and sustainability, help create effective spaces for science and society to dialogue.

## Conclusions

Research capacity is defined as the ability to identify and prioritize problems, build effective institutions and organisations, and identify appropriate solutions to social problems. Thus understood, research capacity entails not just the ability to *produce* knowledge but also to *request* it through the articulation of research agendas and to *use* it through its translation into policy, products, services or practices. Above all, research capacity is necessary to break the cycle of dependency that keeps LMICs subjected to the trajectories of development imagined elsewhere and should be considered a fundamental entitlement ( Mormina, 2019).

Many LMICs are assumed to have weak local research capacity and strong dependency on knowledge created in HICs, and therefore RCD is justified on the need to assist these countries to produce and mobilize knowledge effectively and sustainably. This presupposes a certain directionality in the design of RCD interventions that is highly normative, albeit implicitly, thus obscuring the ethical choices that different actors make about research capacity and the consequences of these decisions for societies.

RCD does not occur in a vacuum but against the backdrop of a centuries-old hegemony of western science that emphasises economic development and neglects non-western knowledge systems and conceptions of wellbeing. One of the legacies of such epistemic erosion is LMICs continuing dependency on HICs for the solution of problems. RCD may lack the overt violence of the imperial project, but it inevitably reproduces and sustains epistemic and power hierarchies through the transfer of technoscientific blueprints of development, which are disguised in an apolitical rhetoric of partnership and ownership.

Significant sums of aid are currently being given to research institutions in LMICs to support development of research capacities and a number of these institutions report benefits from this support. Scientometric analyses also consistently show a slow but steady increase in the volume of scholarship from LMICs, though how much of it is grounded in local epistemologies, rather than reasserting a western locus of enunciation, is unclear. It is also the case that international aid often supports research relevant for those most left behind that the *local* (often construed as a unified actor) would not regard worthy of attention. Given this complex landscape, the effects of RCD cannot be easily categorised as good vs bad, and it should be clear that we are not suggesting that RCD has no relevance in development. We acknowledge and know from a long experience of living and working in LMICs that there are real capacity problems that can only be solved through genuine partnerships. The goal here is rather to alert attention to the problematic nature of technocratic approaches to RCD that falsely claim neutrality. RCD is both political and normative. Research capacity discourses are articulated from a deficit perspective that reasserts western hegemony and imposes monolithic blueprints of excellence to the detriment of alternative epistemologies and knowledge production models. Research capacity processes entail not only technical but also normative and political questions, which this paper has aimed to draw attention to. These must be made transparent by those at the forefront of the practice.

It is not our aim or position to offer specific policy prescriptions. These will vary according to each context. This paper seeks to nuance the conversation and practice around RCD by engaging with critical questions regarding what research capacity actually means for real people. ‘Capacity for what? Capacity for whom?’ Engaging with these questions requires understanding not just how knowledge contributes to positive social change but also how RCD interventions can define what knowledge is produced in the first place, and who benefits from it. The value-centred approach to RCD proposed in this article guided by principles of cognitive justice leading to epistemic pluralism, accessibility underpinned by systems thinking, and sustainability grounded on localisation can serve as a starting point to bring to the fore different voices and perceptions of justice into the design, implementation and evaluation of RCD interventions.

## Data availability

All data underlying the results are available as part of the article and no additional source data are required.
