# Supplementary material for: ‘Capacity for what? Capacity for whom?’ A decolonial deconstruction of research capacity development practices in the Global South and a proposal for a value-centred approach
Source: Wellcome Open Res. 2021 May 26;6:129. [Version 1] doi: 10.12688/wellcomeopenres.16850.1 (PMC8729187; doi:10.12688/wellcomeopenres.16850.1)
Supplement: Peer review history

# Peer review: Reviewer response for version 1

**DOI:** 10.21956/wellcomeopenres.18587.r46439

**Published:** 2022-01-04

**Authors:** Budd Lionel Hall

**Affiliations:** 1 Public Administration, University of Victoria, Victoria, BC, Canada; 2 UNESCO Chair in Community-Based Research, Victoria, BC, Canada

This is a brilliant article which addresses a gap in the decolonial critiques of research and knowledge production. It is a critique of the colonized status and matrices of power that are present in the theory and practice of research capacity development (RCD). They propose a value based approach to RCD based on the principles of cognitive justice, systems thinking and localization. One consideration for the authors would be to replace cognitive justice with the term epistemological justice as 'cognitive' is a term that normally excludes the affective emotional or even spiritual side of knowledge.

Is the work clearly and accurately presented and does it cite the current literature?

Yes

If applicable, is the statistical analysis and its interpretation appropriate?

Yes

Are all the source data underlying the results available to ensure full reproducibility?

Yes

Is the study design appropriate and is the work technically sound?

Yes

Are the conclusions drawn adequately supported by the results?

Yes

Are sufficient details of methods and analysis provided to allow replication by others?

Yes

Reviewer Expertise:

Knowledge democracy, community-based participatory research, social movement learning

I confirm that I have read this submission and believe that I have an appropriate level of expertise to confirm that it is of an acceptable scientific standard.

# Peer review: Reviewer response for version 1

**DOI:** 10.21956/wellcomeopenres.18587.r44071

**Published:** 2021-06-23

**Authors:** Sarah Cummings

**Affiliations:** 1 Knowledge, Technology and Innovation, Wageningen University & Research, Wageningen, The Netherlands

This is a very interesting and topical paper, written by two authors who have an impressive track-record in this area. In this paper, they shift their analysis to research capacity development, focusing on the need to make visible its ‘normative and epistemological dimensions’ (p. 1). Their call for cognitive justice and epistemic pluralism is inspiring.

 Research capacity development is an important area which needs to be addressed in the creation of the imaginary of the knowledge society. The knowledge society started as an academic concept, developed by Drucker and others, but it has been used more recently by governments to create a vision of an emerging future society often linked to technologies, particularly ICTs and economic development (Hornidge, 2011) ^1^ The authors argue convincingly, in my opinion, that research capacity development is often based on the ‘belief about the centrality of scientific knowledge in the development process’ (p. 3) and that this goes hand-in-hand with the dominance of the techno-scientific paradigm. Although the authors emphasize this paradigm, they play less attention to the fact that this is probably the dominant paradigm because it reflects of the economic interests of the governments of the global North (see, for example, Cummings *et al.*, 2018). ^2^ As a consequence of these embedded economic interests, research capacity development is often directed at ‘strengthening the production of knowledge relevant to the knowledge economy’ (p. 5) while this emphasis on the knowledge economy is also enshrined in the Sustainable Development Goals themselves. The authors contend that the dominance of scientific and Western knowledge has its roots in colonialism because the ‘erosion of indigenous knowledge’ was central to the colonial project. This has led to a situation in which there is a lack of intellectual self-confidence in the global South and a concomitant dependency on the knowledge of the global North for the solution of problems. As a result, the global South is the ‘location of problems and backwardness’ while the North is the ‘location of solutions and progress’ (p. 5). The authors then link this approach to the case study of Makerere University, with a donor-funded emphasis on the health sciences, and to the Green Revolution:

 Mechanized farming, large-scale irrigation, chemical fertilizers, and high-yield seed varieties where not technologies designed with the poor in mind: they required access to capital and good quality land, which were only accessible to and controlled by wealthier farmers (Patel, 2013). ^3^

 The authors write in a convincing manner about the role of the apparently ‘rigorous and politically-neutral language’ (p. 5) of research excellence which configures and appraises research capacity in the Global South according to the metrics and standards of the Global North, finding this research capacity insufficient. They also make a plea for a systems thinking to research capacity development, resonating with a forthcoming chapter I have written with colleagues Nyamwaya Munthali and Peter Shapland in which we also argue of the need for a systems approach to, in this case, decolonization of knowledge. This chapter will appear in the new book ‘Politics of knowledge in inclusive development and innovation’ written by colleagues from the Knowledge, Technology and Innovation (KTI) group at Wageningen University & Research (Ludwig *et al.*, In Press). ^4^ While arguing of the need for systems thinking, the authors consider that a historical perspective is needed to complement it. The multi-level perspective to innovation systems, originating from the work of Geels (2002, 2005) ^5^^,^^6^ and others, represents a heuristic of how to bring about transformative societal change, with system changes being driven by change agents, in combination with landscape pressures, regime destabilization processes, and ‘upscaling’ of innovations developed in niches (Wieczorek, 2018). ^7^ The authors rightly argue that this perspective can be improved by adding both a historical and political perspective.

**Some suggestions**

 Although I applaud the authors’ call for a new type of research founded on ‘reflexive commitment to justice and the creation of social value’ (p. 6) that promotes epistemic pluralism and more sustainable knowledge systems, there a few additional nuances that I would like to suggest. First, although the authors are rightly very critical of the current dominant approaches in research capacity development, this focus on the dominant discourse related to research capacity development misses some of the promising, diverse approaches which represent some of the building blocks for a new type of research capacity development of which they are advocates. Many of these are currently in existence in protected niches – using the terminology of the multi-level perspective - such as, for example, WOTRO Science for Global Development, part of the Netherlands Research Organization, or the Swiss Programme for Research on Global Issues for Development (r4d programme). In these spaces and probably others, there is increasing emphasis on local knowledge and local solutions. Second, in the scientific world, there is growing enthusiasm for transdisciplinary research in which the experiential and local knowledge of non-academic actors drives the research agenda for the resolution of real world problems. For example, Valerie Brown and colleagues have integrated multiple knowledges – individual, community, professional and more – in the resolution of complex societal or ‘wicked’ problems (Brown 2011, Brown et al 2010). ^8^^,^^9^ In these processes, ‘transdisciplinary moves to a broader knowledge base have moved on to giving equal respect to the personal, biophysical, social, ethical, aesthetic, sympathetic and reflective domains of knowledge’ (Brown, 2015, 211). ^10^ Third, the authors’ emphasis on the ‘social value’ of research as being defined as products, processes or services (p. 4), might inadvertently also play a role in undermining research as part of the ‘academic imagination’ because the value of research is not only functional but also how it can add to societies’ intangible ability to think and reflect, not only having an impact on their ability to solve complex problems. As Ozbilgin argues in a study of the International Human Resource Management ‘How far does continuing to exclude those regions, which are already underprivileged, from academic imagination contribute to the vicious cycle of their poverty?’ (2004, 216). ^11^

 Finally, the paper moves rather uncomfortably between the terminologies global North and global South, and High Income Countries (HICs) and Low and Middle Income Countries (LMICs). These problematic binaries are characteristic of development-related research, including “ ‘…developing vs developed, industrial vs agrarian, low income vs high income, Third World vs First World, global South vs global North, core vs periphery, sub-Saharan Africa vs North Africa, etc. […] … … These binaries shackle us, they do not liberate” ’ (Pailey 2020: 734). ^12^ Horner (2020) ^13^ and others consider that ‘global development’ should be the new paradigm to replace international development, recognizing that “‘we are all developing countries now”’ (Raworth, 2018). ^14^ In this context, the authors might consider using ‘formerly colonized countries’ instead of global South and LMICs, and ‘Western donor countries’ instead of the global North and HICs. Although these are still binaries, these terminologies better call attention to the continuous impact of colonialism and, at the same time and as others have argued, disrupt development’s effort to conceal its colonial roots.

**Reflections on the methodology of an open peer review**

 This is the first time that I have written a peer review in such an open process, and it is I think it is probably the way forward, particularly when I often have considerable suspicions of the identity of the author in a traditional blinded review process. This new review process – when combined with open access to the paper for review – probably calls for a more appreciative review, something which, for example, the community-led, open access Knowledge Management for Development has long appreciated. However, I also note that it encourages a more reflexive review in which I have focused on what the paper does to my own understanding at the same time as reflecting on how it fits within the field, rather than the more overt focus on how the paper can be ‘improved’, characteristic of a more traditional double-blind peer review process. In this case, my main inclination is to applaud from the side-lines and I can think of no direct means of improvement: it is an interesting, well-written and well-argued paper. As you can see from the review above, I mostly agree with their analysis, although there are a few small areas where I think a slightly more nuanced reflection would possible.

Is the work clearly and accurately presented and does it cite the current literature?

Yes

If applicable, is the statistical analysis and its interpretation appropriate?

Not applicable

Are all the source data underlying the results available to ensure full reproducibility?

No source data required

Is the study design appropriate and is the work technically sound?

Yes

Are the conclusions drawn adequately supported by the results?

Yes

Are sufficient details of methods and analysis provided to allow replication by others?

Yes

Reviewer Expertise:

NA

I confirm that I have read this submission and believe that I have an appropriate level of expertise to confirm that it is of an acceptable scientific standard.

## References

[1] : ‘Knowledge Society’ as Academic Concept and Stage of Development: A Conceptual and Historical Review.2011; 10.1142/9789814343688_0004 87-127 10.1142/9789814343688_0004

[2] : Critical discourse analysis of perspectives on knowledge and the knowledge society within the Sustainable Development Goals. *Development Policy Review* .2018;36(6) : 10.1111/dpr.12296 727-742 10.1111/dpr.12296

[3] : The Long Green Revolution. *Journal of Peasant Studies* .2013;40(1) : 10.1080/03066150.2012.719224 1-63 10.1080/03066150.2012.719224

[4] : The politics of knowledge in inclusive development and innovation. *Routledge* .2022;

[5] : Technological transitions as evolutionary reconfiguration processes: a multi-level perspective and a case-study. *Research Policy* .2002;31(8-9) : 10.1016/S0048-7333(02)00062-8 1257-1274 10.1016/S0048-7333(02)00062-8

[6] : Processes and patterns in transitions and system innovations: Refining the co-evolutionary multi-level perspective. *Technological Forecasting and Social Change* .2005;72(6) : 10.1016/j.techfore.2004.08.014 681-696 10.1016/j.techfore.2004.08.014

[7] : Sustainability transitions in developing countries: Major insights and their implications for research and policy. *Environmental Science & Policy* .2018;84: 10.1016/j.envsci.2017.08.008 204-216 10.1016/j.envsci.2017.08.008

[8] : Multiple knowledges, multiple languages: are the limits of my language the limits of my world?. *Knowledge Management for Development Journal* .2010;6(2) : 10.1080/19474199.2010.532148 120-131 10.1080/19474199.2010.532148

[9] : Tackling Wicked Problems : Through the Transdisciplinary Imagination. *Routledge* .2010;

[10] : Utopian thinking and the collective mind: Beyond transdisciplinarity. *Futures* .2015;65: 10.1016/j.futures.2014.11.004 209-216 10.1016/j.futures.2014.11.004

[11] : “International” human resource management. *Personnel Review* .2004;33(2) : 10.1108/00434804105180559 205-221 10.1108/00434804105180559

[12] : De‐centring the ‘White Gaze’ of Development. *Development and Change* .2020;51(3) : 10.1111/dech.12550 729-745 10.1111/dech.12550

[13] : Towards a new paradigm of global development? Beyond the limits of international development. *Progress in Human Geography* .2020;44(3) : 10.1177/0309132519836158 415-436 10.1177/0309132519836158

[14] : Doing the Doughnut at the G20?. *Blog* .2018; Reference source
